# Beneficial effects of Korean red ginseng on lymphocyte DNA damage, antioxidant enzyme activity, and LDL oxidation in healthy participants: a randomized, double-blind, placebo-controlled trial

**DOI:** 10.1186/1475-2891-11-47

**Published:** 2012-07-17

**Authors:** Ji Young Kim, Ju Yeon Park, Hee Jung Kang, Oh Yoen Kim, Jong Ho Lee

**Affiliations:** 1 Department of Culinary Nutrition, Woosong University, Daejeon, Korea; 2 Division of Metabolic Diseases, Center for Biomedical Science, Korea National Institute of Health, Chungbuk, Korea; 3 Department of Food and Nutrition, Research Laboratory for Clinical Nutrigenetics/Nutrigenomics, Yonsei University, Seoul, Korea; 4 Department of Food Science and Nutrition, Dong-A University, Busan, Korea; 5 Department of Food and Nutrition, Brain Korea 21 Project, College of Human Ecology, Yonsei University, Seoul, Korea; 6 Department of Food and Nutrition, Yonsei University, 134 Shinchon-Dong, Sudaemun-Gu, Seoul, 120-749, Korea

**Keywords:** Antioxidant enzymes, Korean red ginseng, LDL oxidation, Lymphocyte DNA damage, Oxidative stress

## Abstract

**Background:**

The reported health benefits of Korean red ginseng (KRG) include antioxidant, antitumor, antimutagenic, and immunomodulatory activities; however, the effects on oxidative stress have not yet been evaluated. Therefore, we assessed the effect of KRG on antioxidant enzymes and oxidative stress markers in humans.

**Methods:**

We conducted a randomized, double-blind, placebo-controlled study with three groups, including placebo, low-dose (3 g/day), and high-dose (6 g/day), which were randomly assigned to healthy subjects aged 20–65 years. Lymphocyte DNA damage, antioxidative enzyme activity, and lipid peroxidation were assessed before and after the 8-week supplementation.

**Results:**

Fifty-seven subjects completed the protocol. Plasma superoxide dismutase (SOD) activity after the 8-week KRG supplementation was significantly higher in the low-and high-dose groups compared to baseline. Plasma glutathione peroxidase (GPx) and catalase activities were also increased after the high-dose supplementation. Furthermore, the DNA tail length and tail moment were significantly reduced after the supplementation (low-dose and high-dose), and plasma oxidized low-density lipoprotein (LDL) levels were reduced in low-dose and high-dose groups, but increased in the placebo group. The net changes in oxidized LDL after the supplementation differed significantly between both KRG supplementation groups and the placebo group. Net changes in GPx, SOD and catalase activities, and DNA tail length and tail moment were significantly different between the high-dose group and the placebo group. Additionally, the net changes in urinary 8-epi-PGF_2α_ were significantly different between the KRG supplementation groups and the placebo group.

**Conclusions:**

KRG supplementation may attenuate lymphocyte DNA damage and LDL oxidation by upregulating antioxidant enzyme activity.

## Background

Oxidative stress describes a set of intracellular or extracellular conditions that lead to the chemical or metabolic generation of reactive oxygen/nitrogen species, including superoxide radicals, hydrogen peroxide (H_2_O_2_), hydroxyl radicals, singlet oxygen, lipid hydroperoxides, peroxynitrite, and related species [1]. Reactive oxygen/nitrogen species can cause oxidative damage to essential cellular constituents, such as membrane lipids, proteins, and DNA, which may ultimately result in cell death [2]). Aerobic organisms attempt to protect themselves against oxidative damage with exogenous antioxidants (e.g., vitamin E, ascorbic acid, and carotenoids) obtained through the diet as well as endogenous antioxidants (e.g., glutathione, glutathione peroxidase (GPx), catalase, and superoxide dismutase (SOD)). Furthermore, oxidative damage mediated by the reactive oxygen species listed above is known to contribute to the aging process and the pathogenesis of cancer, cardiovascular disease, and other degenerative diseases [3].

Ginseng (*Panax ginseng* C.A. Meyer) is a perennial herb belonging to the *Panax* family (Araliaceae). Its roots are used as a general tonic in traditional oriental medicine to increase health, longevity, and vitality, especially in the elderly [4]. White Ginseng is known to exert a number of effects, such as suppressing the growth and metastasis of melanoma (B16) [5] and activating GPx and SOD to protect against free radical damage [6]. Interest in Korean red ginseng (KRG) has grown recently, as it is one of the foods that can easily be consumed daily in Korea. Red ginseng is produced by steaming and drying fresh ginseng, a process that chemically transforms the components and alters their biological properties [7]. The non-saponin components of red ginseng are thought to improve learning and memory [8], while acid polysaccharides activate natural killer cells and stimulate interferon production [9]. Mochizuki et al. [10] reported that 20(R)- and 20(S)-ginsenoside-Rg3, the ginseng saponins, possess the ability to inhibit the lung metastasis of cancer by inhibiting the invasion and adhesion of tumor cells. According to Kubo and Tong [11], red ginseng extract influenced tumor cell lysosomes by promoting the uptake of mitomycin C, which has cytocidal effects. Kim et al. [12] also found that ginseng extracts scavenged 40% of hydroxyl radicals at 0.1 mg/ml and completely scavenged superoxide radicals at 2 mg/ml. In addition, KRG was also shown to affect immunological markers in individuals with human immunodeficiency virus [13]. Although a number of pre-clinical studies have reported medicinal benefits of KRG including antioxidant, antitumor, antimutagenic [10-12] and a clinical study has reported immunomodulatory actions [13], the effects of KRG on oxidative stress and lipid peroxidation have not been properly evaluated in healthy subjects. Thus, we conducted a randomized, double-blind, placebo-controlled clinical trial in healthy subjects to evaluate the effects of KRG supplementation on biomarkers of oxidative stress and lipid peroxidation, which included lymphocyte DNA damage, urinary 8-epi-prostaglandin F_2α_ (PGF_2α_), plasma oxidized low-density lipoprotein (LDL), and antioxidant enzymes.

## Methods

### Subjects

Sixty-nine healthy subjects (aged 20–65 years) were recruited during routine check-ups at a health promotion center at Yonsei University Hospital from May to November in 2009. Participants were asked to visit four times (1 week before and at 0, 4, and 8 weeks). Blood samples were taken at weeks 0 and 8, and diet and capsule intakes were assessed at weeks 0, 4, and 8. Healthy subjects who either used cigarettes or consumed alcohol (or both) were enrolled in this study at the Laboratory of Clinical Nutrigenetics/Nutrigenomics at Yonsei University. The exclusion criteria were the consumption of more than three servings of vegetables or fruit per day, antioxidant or vitamin/mineral supplementation, history of chronic disease (e.g., diabetes, heart disease, renal disease), or the use of medication (e.g., lipid-lowering or antihypertensive medications, anti-platelets, or disease-related medications). The participants were interviewed to determine their smoking and drinking habits. Smoking status was categorized as “current smoker” or “nonsmoker,” and alcohol consumption was categorized as “current drinker” or “nondrinker.” Nonsmokers or nondrinkers included both ex-consumers and those who had never consumed. Ex-smokers and ex-drinkers were defined as subjects who stopped smoking or drinking at least 1 year prior to participating in the study. Current smokers were asked the number of cigarettes smoked per day. Current drinkers were asked about the type and amount of alcohol consumed per day, and the amount of alcohol was then calculated as grams per day.

The study subjects number calculation assumed a two-tailed alpha = 0.05 and 1-β = 90% to detect a 10% difference with a standard error of 0.28 in the comet assay which is primary outcome and an attrition rate (20%). Biomarkers for testing antioxidant/oxidative effects and the number of subjects were determined by following the criteria recommended by the Korea Food and Drug Administration. Of the 69 study subjects, 12 subjects (placebo group, *n* = 4; low-dose group [3 g KRG/day], *n* = 4; high-dose group, [6 g KRG/day], *n* = 4) discontinued the study for personal reasons. No adverse reactions (e.g., fever, hot flush, nausea, vomiting, diarrhea) due to KRG supplementation were observed among the 57 subjects who completed the study.

### Test capsule and study design

Identical-looking capsules contained red ginseng (low dose, 300 mg; high dose, 600 mg) or placebo (300 mg of KRG-flavored capsule containing corn starch). Red ginseng and placebo capsules were provided by the Korea Ginseng Cooperation (KGC, Daejeon, Korea). The red ginseng contained 16.58 mg/g total ginsenosides, and the ratio of protopanaxadiol ginsenosides (Rb1, Rb2, Rc, Rd, and Rg3) to protopanaxatriol ginsenosides (Rg1, Re, and Rf) was 1.65. Analyses of common ginsenosides were performed in quadruplicate using standard HPLC-UV techniques [14] at the Korean Ginseng Research Institute in Daejeon, Korea.

This study design was the randomized double-blind, placebo-controlled intervention trial for 8 week and was approved by the Institutional Review Board of Yonsei University (#RA-2009-650). After written informed consent was obtained, subjects were randomly assigned to placebo (n = 23) or KRG (low-dose [n = 24] or high-dose [n = 22]) groups. All subjects were asked to take 10 capsules in total per day immediately after any main meals. For example, three capsules after breakfast, three capsules after lunch, and four capsules after dinner. Compliance of KRG consumption was assessed at the end of the study by counting capsules remaining and self-recording. If compliance was beneath 75%, the subject dropped out. All participants were encouraged to maintain their usual lifestyle and dietary habits.

### Assessment of dietary intake and level of physical activity

The subjects’ usual diet information was obtained using both a 24-hour recall method and a semi-quantitative food frequency questionnaire (SQFFQ), which has been tested previously for validity [15]. We used the former to carry out analyses and the latter to check if the data collected by the 24-hour recall method was representative of the usual dietary pattern.

All the subjects were given written and verbal instructions by a registered dietitian for the completion of a 3-day (two weekdays and one weekend) dietary record every 4 weeks. On the sheet, the subjects were instructed to record the amount of food before ingestion and any remaining after ingestion by weighing the foods. One week before the start of the study, all the participants were advised to continue their usual diet for a week and were instructed to complete a 3-day dietary record as the baseline measurement, which was compared with the record obtained by 24-hour recall methods at a previous visit to check their records. The participants were also instructed to record their physical activity in a diary for 24 hours every 4 weeks. To check participants’ compliance during the whole study period, the dietitian interviewed them biweekly by telephone. During the study period, all participants were also encouraged to maintain their usual lifestyle.

Dietary energy values and nutrient content from the 3-day food records were calculated using the Computer Aided Nutritional analysis program (CAN-pro 2.0, Korean Nutrition Society, Seoul, Korea). Total energy expenditure (kcal/day) was calculated from activity patterns, including basal metabolic rate, physical activity for 24 hours [16], and specific dynamic action of food. Basal metabolic rates for each subject were calculated with the Harris-Benedict equation [17].

### Anthropometric parameters, blood pressure, and blood and urine collection

Body weight and height were measured without clothes or shoes in the morning to determine the body mass index (BMI; kg/m^2^). Blood pressure was measured with an automatic blood pressure monitor (TM-2654, A&D, Tokyo, Japan) in triplicate using the left arm of a seated subject after a 20-min rest. After a 12 h fasting period, venous blood specimens were collected in EDTA-treated and plain tubes, centrifuged to produce plasma or serum, and stored at −70°C until analysis. Urine was collected in polyethylene bottles containing 1% butylated hydroxytoluene after 12 h of fasting. The tubes were immediately covered with aluminum foil and stored at −70°C until analysis.

### Serum lipid profile and fasting glucose

Fasting total cholesterol and triglycerides were measured using commercially available kits on a Hitachi 7150 Autoanalyzer (Hitachi Ltd., Tokyo, Japan). After precipitation of chylomicrons with dextran sulfate magnesium, concentrations of LDL and high-density lipoprotein (HDL) cholesterol in the supernatants were determined enzymatically. In participants with serum triglyceride concentrations below 400 mg/mL, LDL cholesterol was indirectly measured using the Friedewald formula. Fasting glucose levels were determined by the glucose oxidase method using a Beckman glucose analyzer (Beckman Instruments, Irvine, CA, USA).

### Safety parameters

Serum Blood Urea Nitrogen (BUN) and creatinine concentrations were measured by a colorimetric method using commercially available kits on a Hitachi 7180 Autoanalyzer (Hitachi Ltd., Tokyo, Japan). Serum aspartate aminotransferase (AST) and alanine aminotransferase (ALT) were measured by a kinetic UV method based on recommendations by the International Federation of Clinical Chemistry (IFCC) using commercially available kits on a Hitachi 7180 Autoanalyzer (Hitachi Ltd., Tokyo, Japan). A complete blood count (CBC) test was determined using the HOROBA ABX diagnostic (HORIBA ABX SAS, Parc Euromedicine, France).

### Alkaline comet assay for DNA damage

As a primary outcome measure, DNA damage was assessed using the comet method described by Green et al. [18]. Whole blood was mixed with phosphate buffered saline and poured gently over a lymphocyte separation solution (Histopaque-1077, Sigma Chemical Co., Korea). After centrifugation at 1,450 rpm for 4 min, lymphocytes were transferred to another tube, mixed with 0.7% low-melting agarose, and then added to slides pre-coated with 0.5% agarose. The slides were immersed in freshly prepared cold lysing solution and subjected to electrophoresis. The slides were then washed with neutralizing buffer and treated with ethanol. Tail length (μm) and tail moment (% of DNA in the tail × tail length) were evaluated using image analysis software (Komet 5.0, Kinetic Imaging, UK) and fluorescence microscopy (Leica, Germany) equipment with filters. Images from 50 cells from each slide were analyzed. All steps were performed in dim light, and the electrophoresis tank was covered with black paper to avoid additional light-induced DNA damage.

### Antioxidative enzyme activities

Plasma and erythrocyte antioxidative enzyme activities were measured as secondary outcomes. Plasma and erythrocyte SOD activity was determined based on the generation of superoxide radicals produced by xanthine and hypoxanthine. Superoxide radicals react with tetrazolium chloride salts to produce a red formazan dye. SOD activity was estimated by the inhibition of this colorimetric reaction using an assay kit (Cayman Chemical, Ann Arbor, MI, USA), and the absorbance at 450 nm was measured with a Wallac Victor^2^ multilabel counter (Perkin Elmer Life Sciences, Turka, Finland). The intra-assay and inter-assay coefficients of variance were 4.43% and 8.26%, respectively. Plasma GPx activity was indirectly measured using an assay kit that involved a coupled reaction with glutathione reductase (Cayman Chemical). Oxidized glutathione, generated by the reduction of hydroperoxide by GPx, was restored to its reduced state by glutathione reductase and nicotinamide adenine dinucleotide phosphate (NADPH). NADPH oxidation was monitored at a lower 340 nm absorbance, which was measured with the Wallac Victor^2^ multilabel counter. The intra-assay and inter-assay coefficients of variance were 6.01% and 7.68%, respectively. Plasma and erythrocyte catalase activity was measured based on the generation of formaldehyde from methanol in the presence of H_2_O_2_ using an assay kit (Cayman Chemical). Formaldehyde forms a bicyclic heterocycle with the chromogen Purpald, which changes from colorless to a purple color when oxidized. The absorbance was measured at 540 nm with the Wallac Victor^2^ multilabel counter. The intra-assay and inter-assay coefficients of variance were 5.52% and 6.78%, respectively.

### Plasma oxidized LDL concentration and Urinary 8-epi-PGF_2α_

As one of the secondary outcome measures, plasma oxidized LDL concentration and urinary 8-epi-PGF_2α_ excretion. Plasma oxidized LDL was determined with an enzyme immunoassay (Mercodia, Uppsala, Sweden), and the color reaction was measured at 450 nm with the Wallac Victor^2^ multilabel counter. The intra-assay and inter-assay coefficients of variance were 6.52% and 8.15%, respectively. Urinary 8-epi-PGF_2α_ was measured using a colorimetric enzyme immunoassay (Bioxytech urinary 8-epi-PGF_2α_ assay kit; OXIS International Inc., OR, USA). The resulting color was measured at 650 nm using the Wallac Victor^2^ multilabel counter. Urinary creatinine was determined using a Hitachi 7180 Autoanalyzer (Hitachi Ltd., Tokyo, Japan) by the alkaline picrated (Jeffe) reaction. Urinary 8-epi-PGF_2α_ concentrations were expressed as pmol/mmol of creatinine. The intra-assay and inter-assay coefficients of variance were 4.32% and 9.57%, respectively.

### Statistical analysis

Statistical analyses were performed with SPSS version 12.0 for Windows (Statistical Package for the Social Sciences (SPSS) Inc., Chicago, IL, USA). We used the Wilcoxon signed-rank test to determine within-group differences of biochemical parameters before (baseline) and after the intervention. The nonparametric Kruskal-Wallis test and analysis of covariance (ANCOVA) using the general linear model (GLM) with adjustments for age, sex, BMI, smoking, drinking, systolic BP, diastolic BP, and baseline values were used to compare the three groups. Spearman’s correlation coefficients were used to determine relationships between variables. After determining whether data was normally distributed by the Kolmogorov-Smirnov and Shapiro-Wilk W tests, a logarithmic transformation was applied to non-normal data before statistical analysis. For descriptive purposes, the results are expressed as untransformed and unadjusted mean values. Continuous variables are expressed as mean ± SEM, and categorical variables are expressed as absolute numbers and percentages. A two-tailed value of *P* less than 0.05 was considered significant.

## Results

### Baseline clinical characteristics and dietary intake

Table 1 shows the baseline clinical characteristics of the study subjects. Anthropometric and biochemical parameters before treatment were not significantly different among the groups. In addition, KRG supplementation had no significant effect on BMI and blood pressure. After treatment, serum lipid profiles (e.g., total cholesterol, triglyceride, HDL- and LDL-cholesterol) and safety parameters (e.g., liver and kidney function tests, complete blood count) did not differ among groups (data not shown). In addition, no significant differences were observed for daily nutrient intake before and after the intervention in all the groups (Table 2).

**Table 1:** Anthropometric parameters and blood pressure before and after intervention

	**Placebo (n = 19)**	**Low-dose (n = 19)**	**High-dose (n = 19)**	***P***^***a)***^			
Age (yr)	37.0 ± 2.24	36.4 ± 1.73	35.3 ± 1.68	0.910			
Male / Female (n)	7/12	7/12	9/10	0.747			
Body mass index (BMI; kg/m^2^)							
Pre-treatment	23.4 ± 0.60	24.0±0.93	23.5±0.70				
Post-treatment	23.6 ± 0.67	24.3±0.93	23.6±0.65				
Change	0.27±0.16	0.26±0.15	0.11±0.14	0.850			
Cigarette smoker, n (%)	8 (42.1)	6 (31.6)	8 (42.1)	0.948			
Alcohol drinker, n (%)	15 (78.9)	13 (68.4)	13 (68.4)	0.706			
Blood pressure (mmHg)							
Systolic blood pressure (mmHg)							
Pre-treatment	115.3±2.92	118.8±3.22	117.5±2.32				
Post-treatment	114.7±2.08	118.8±3.10	118.2±1.94				
Change	−0.55±1.97	−0.03±0.19	0.74±1.65	0.740			
Diastolic blood pressure (mmHg)							
Pre-treatment	76.6±1.83	80.6±1.98	78.4±1.81				
Post-treatment	75.6±1.93	77.3±2.14^*^	76.2±1.71				
Change	−0.92±1.88	−3.29±1.28	−2.16±1.75	0.599			

Results are expressed as mean ± standard error of the mean (SEM) or n (%).

Baseline values for the placebo and test groups were not significantly different (nonparametric Kruskal-Wallis test).

^*^P < 0.05 compared with the baseline in each group (Wilcoxon signed-rank test).

^a)^ Evaluated by nonparametric Kruskal-Wallis test.

**Table 2:** Daily energy expenditure and nutrient intake before and after intervention

	**Placebo (n = 19)**	**Low-dose (n = 19)**	**High-dose (n = 19)**	***P***^***a)***^
Total energy expenditure (kcal)				
Pre-treatment	2329.3±78.9	2331.8±90.5	2299.5±57.6	
Post-treatment	2268.3±76.7	2273.2±98.1	2300.0±68.1	
Change	−61.0±36.9	−58.6±33.6	0.50±32.9	0.317
Estimates of dietary nutrient intake				
Total calorie intake (kcal)				
Pre-treatment	1789.2±79.6	1726.4±104.4	1847.9±114.2	
Post-treatment	1892.9±72.9	1820.1±81.9	1966.3±97.8	
Change	103.7±79.0	93.7±69.1	118.4±75.1	0.991
Carbohydrate (%)				
Pre-treatment	60.0±1.57	60.8±1.09	62.4±0.92	
Post-treatment	61.2±1.66	59.9±1.72	61.1±1.34	
Change	1.16±1.78	−0.94±1.78	−1.28±1.44	0.767
Protein (%)				
Pre-treatment	16.0±0.61	16.3±0.62	15.8±0.42	
Post-treatment	16.5±0.75	16.0±0.61	15.4±0.53	
Change	0.56±0.86	−0.36±0.75	−0.38±0.67	0.280
Fat (%)				
Pre-treatment	24.4±1.47	23.9±1.05	22.4±1.07	
Post-treatment	22.7±1.79	24.3±1.55	22.9±1.14	
Change	−1.67±1.65	0.43±1.22	0.50±1.22	0.733
TEE/TCI				
Pre-treatment	1.37±0.11	1.43±0.09	1.34±0.09	
Post-treatment	1.23±0.06	1.29±0.08	1.23±0.07	
Change	−0.14±0.10	−0.14±0.07	−0.12±0.07	0.990

Results are expressed as standard error of the mean (SEM).

No significant differences in the week 0 value between the placebo and test groups were found by nonparametric Kruskal-Wallis test.

^a)^ Evaluated by nonparametric Kruskal-Wallis test.

### Antioxidant enzyme activities

Plasma SOD activity after the 8-week KRG supplementation was significantly higher than baseline activity (low-dose; *P* = 0.038, high-dose; *P* = 0.016), and the net changes were significantly different between the high-dose group and the placebo group (*P* = 0.021) after adjusting for age, sex, BMI, smoking, drinking, systolic BP, diastolic BP, and baseline values (Table 3). The plasma GPx and catalase activities after the 8-week high-dose supplementation were higher than baseline (*P* = 0.006 and 0.016, respectively), and the net changes also were significantly different between the high-dose group and the placebo group (*P* = 0.032 and 0.019, respectively) after adjustment. Erythrocyte SOD and catalase activities were also measured. No significant differences were observed in the baseline activities of both enzymes among the three groups (SOD: placebo, 44.1 ± 3.14; low-dose, 46.0 ± 2.93; high-dose; 42.3 ± 2.99; Catalase: placebo, 231.1 ± 1.91; low-dose, 224.3 ± 3.48; high-dose; 223.7 ± 2.78). After the eight weeks, the activities of both enzymes were significantly increased in the high-dose group (p < 0.05). In particular, the net changed values for erythrocyte SOD activities were significantly different among the three groups after the adjustment (SOD: placebo, -3.58 ± 2.39; low-dose, -0.07 ± 1.81; high-dose; 3.50 ± 1.95, p = 0.030; Catalase: placebo, -6.74 ± 2.94; low-dose, 1.21 ± 3.45; high-dose; 4.46 ± 2.55, p = 0.120).

**Table 3:** Antioxidant enzyme activity and lipid peroxidation before and after intervention

	**Placebo (n = 19)**	**Low-dose (n = 19)**	**High-dose (n = 19)**			
SOD activity (U/ml)						
Pre-treatment	12.6±0.97	10.1±0.68	11.9±0.98			
Post-treatment	12.2±0.96	12.1±1.03^*^	14.2±1.15^*^			
Change	−0.46±0.57	1.95±0.80	2.24±0.86^†^			
GPx activity (nmol/min/ml)						
Pre-treatment	41.1±3.71	34.3±1.88	34.9±2.53			
Post-treatment	39.1±3.72	37.4±2.27	43.5±2.53^**^			
Change	−2.00±2.08	3.05±2.15	8.66±2.63^†^			
Catalase (nmol/min/ml)						
Pre-treatment	103.2±11.5	94.5±8.56	86.9±11.7			
Post-treatment	99.0±13.2	118.3±11.8	135.5±12.3^**^			
Change	−4.17±12.4	23.8±12.4	48.5±12.3^†^			
8-epi-PGF_2α_ (pg/mg creatinine)^a)^						
Pre-treatment	1450.3±103.7	1519.0±222.3	1516.3±105.9			
Post-treatment	1641.6±136.5	1323.4±106.6	1383.5±120.2			
Change	191.3±107.7	−195.6±147.6^†^	−132.8±127.9^†^			

SOD, superoxide dismutase; GPx, glutathione peroxidase; 8-epi-PGF_2α_, 8-epi-prostaglandin F_2α_.

^a)^Tested after logarithmic transformation.

Results are expressed as standard error of the mean (SEM).

Tested by Wilcoxon signed-rank test or analysis of covariance (ANCOVA) using a general linear model (GLM) with adjustments for age, sex, BMI, smoking, drinking, systolic BP, diastolic BP, and baseline values.

^*^P <0.05,^**^P <0.01 compared with the baseline in each group; ^†^*P* <0.05, ^††^*P* <0.01 compared with the placebo group.

### Lymphocyte DNA damage

**Figure 1:**
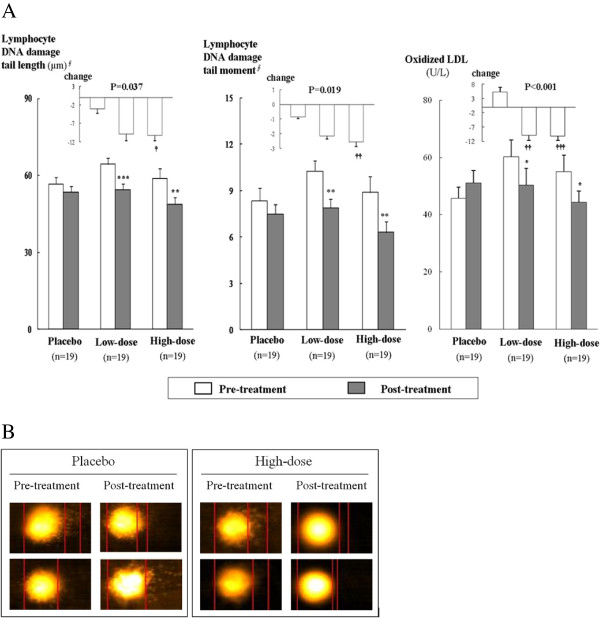
**Effects of 8-week supplementation of Korean red ginseng (KRG) on lymphocyte DNA damage LDL oxidation. A.** Comparison of mean values for damaged tail length (μm) and tail moment in lymphocyte DNA and the concentrations of oxidized LDL (U/L). **B**. Images of lymphocyte DNA damage performed by comet assay before and after treatment between the placebo and high-dose groups. ^∮^Tested after log transformation. *P* values represent significant differences by analysis of covariance (ANCOVA) using a general linear model (GLM) with adjustments for age, sex, BMI, smoking, drinking, systolic BP, diastolic BP, and baseline values. ^*^P <0.05,^**^P <0.01, and ^***^P <0.001 compared with the baseline in each group; ^†^*P* <0.05, ^††^*P* <0.01 , ^†††^*P* <0.001 compared with the placebo group.

The DNA tail length significantly decreased after KRG supplementation compared to baseline (low-dose; *P* = 0.004, high-dose; *P* = 0.003; Figure 1). The tail moment was also significantly reduced by KRG supplementation (low-dose; *P* = 0.012, high-dose; *P* = 0.001). The net changes in tail length and tail moment differed significantly between the high-dose group and the placebo group (*P* = 0.032 and *P* = 0.019, respectively) after adjustment. The images showing lymphocyte DNA damage, as performed by comet assay before and after the treatment, between the placebo and high-dose groups are presented in Figure 1.

### Oxidative stress biomarkers, oxidized LDL, and urinary 8-epi-PGF_2α_

After 8 weeks, the plasma oxidized LDL levels decreased in the low-dose (*P* = 0.003) and high-dose (*P* = 0.001) groups, but increased in the placebo group (*P* = 0.035). The net changes differed significantly between the KRG supplementation groups and the placebo group (vs. low-dose, *P* = 0.001; vs. high-dose, *P <*0.001) after adjustment (Figure 1). We also found significant differences in the net changes of urinary excretion of 8-epi-PGF_2α_ between the KRG supplementation groups and the placebo group (vs. low-dose, *P* = 0.033; vs. high-dose, *P =* 0.049) after adjustment (Table 3).

### Relationship between changes in oxidative stress markers and catalase activity

Among all subjects, changes in oxidized LDL from baseline correlated positively with changes in urinary 8-epi-PGF_2α_ (*r* = 0.305, *P* = 0.030), negatively with changes in catalase activity (*r* = −0.419, *P* = 0.002), and appeared to correlate positively with changes in DNA tail moment (*r* = 0.231, *P* = 0.096); however, this correlation was not significant. After adjusting for sex and age, changes in oxidized LDL correlated positively with both changes in urinary 8-epi-PGF_2α_ (*r* = 0.332, *P* = 0.026) and DNA tail moment (*r* = 0.295, *P* = 0.049) and correlated negatively with changes in catalase activity (*r* = −0.401, *P* = 0.006).

## Discussion

It has been reported that about 200 substances, such as ginsenosides, polysaccharides, polyacetylenes, peptides, and amino acids etc, have been isolated from Korean ginseng, and more than 100 substances have also been isolated from American ginseng and Notoginseng [19,20]. Among the substances isolated from ginseng, ginsenosides are the primary active components of ginseng [21]. According the recent report, more than 60 ginsenosides have been found in the American ginseng [22]. These ginsenosides have been discovered continuously in the Korean ginseng, and it has been assumed that Korean ginseng contains over 60 ginsenosides [20]. Furthermore, Korean ginseng contains more primary non-saponin compounds, phenol compounds, acid polysaccharides, and polyethylene compounds than American ginseng or Sanchi ginseng [23]. KRG is produced by a special process of steaming and drying fresh Korean ginseng. The steaming process hydrolyzes and converts ginsenosides into other types of ginsenosides, such as red ginseng-specific compounds with anticancer properties (ginsenoside-Rh2, -Rh4, -Rs3, -Rs4, -Rg5) and a compound with anti-metastasis and vasodilation properties (ginsenoside-Rg3). The anti-oxidative and anti-cancer activities of KRG appear to be superior to those of white ginseng [21,24].

The reduction in oxidative DNA damage after KRG supplementation observed in the present study is consistent with findings from a previous study using a rat model. Park et al. [25] reported that red ginseng extract inhibited the breaking of *Escherichia coli* ColE1 plasmid DNA as well as the nuclear DNA of rat hepatocytes damaged by oxidative stress. In the present study, 8 weeks of KRG supplementation (6 g/day) significantly decreased DNA tail length (μm) and tail moment, indicators of lymphocyte DNA damage, compared to placebo. Zhang et al. [26] also reported in mice that a 2-day oral administration of 20(S)-ginsenoside Rg3 (20 mg/kg per day) significantly inhibited cyclophosphamide-induced DNA damage in bone marrow cells and peripheral lymphocytes.

Oxidative stress results from an imbalance between antioxidants and reactive oxygen species, such as superoxide anions, H_2_O_2_, and hydroxyl radicals [27]. High levels of reactive oxygen species damage biomolecules (nucleic acids, proteins, and lipids) and generate oxidized LDL, which upregulates lectin-like oxidized low-density lipoprotein receptor-1 in endothelial cells, thereby inducing the generation of more reactive oxygen species through an unclear intracellular feedback mechanism [28]. The antioxidant enzyme SOD eliminates superoxide anions by producing H_2_O_2_ and is the first line of defense against reactive oxygen species toxicity. Furthermore, the enzymes catalase and GPx convert harmful H_2_O_2_ to harmless H_2_O [3,29]. Thus, boosting the activity of these three main antioxidant enzymes by KRG supplementation may decrease oxidative stress.

In the present study, plasma SOD, catalase, GPx, and erythrocyte SOD activities were significantly higher in subjects who received KRG supplementation than in subjects who received the placebo. In the past, the antioxidative effects of KRG have primarily been demonstrated in animal models or *in vitro* studies. Kim et al. [30] reported that, in mice subjected to paraquat-mediated oxidative stress, SOD, catalase, and GPx levels in liver tissue were significantly increased by red ginseng saponin supplementation. In a separate publication, Kim et al. [31] demonstrated that ginsenoside Rh2, a characteristic ginsenoside of red ginseng, is a particularly important active compound that significantly boosts catalase activity. Similarly, Chang et al. [32] reported that the Rb2 subfraction of panaxadiol ginsenosides appeared to strongly induce SOD1 and catalase, which maintain cell viability by lowering the level of oxygen radicals generated by the intracellular metabolism.

The limitations of our study include a small sample size, which renders us unable to generalize our study findings to other populations. We were also limited by the short study duration, and the mechanisms of action were not addressed in the study design. The important findings presented in this study will require further investigation in larger trials that will be carefully designed to include the optimal dose and form of KRG intervention, the study duration, and subject characteristics. In addition, enzyme mass and gene and protein expression levels should be measured to determine whether the increased enzyme activity is due to an increase in protein mass or through the elevation of the specific activities of the enzyme.

## Conclusions

In this randomized, double-blind, placebo-controlled study, KRG supplementation improved biomarkers of oxidative stress*,* as evidenced by decreased plasma oxidized LDL, attenuated lymphocyte DNA damage and increased plasma antioxidant enzyme activity in healthy participants (20–65 years old). In addition, changes in plasma oxidized LDL correlated positively with the decreases in oxidative DNA damage and urinary 8-epi-PGF_2α_ and negatively with changes in catalase activity. Larger prospective studies with a longer intervention period (3 or 6 months) will be necessary to further characterize the beneficial antioxidative activity of KRG and its mechanism of action.

## Abbreviations

GPx, Glutathione peroxidase; SOD, Superoxide dismutase; KRG, Korean red ginseng; PGF2α, 8-epi-prostaglandin F2α; LDL, Low-density lipoprotein; HDL, High-density lipoprotein; AST, Serum aspartate aminotransferase; ALT, Alanine aminotransferase; BUN, Blood urea nitrogen; CBC, Complete blood count; NADPH, Nicotinamide adenine dinucleotide phosphate.

## Competing interests

The authors declare that they have no competing interests.

## Authors’ contributions

JYK and JHL conceptualized this study, and JHL was the Principal Investigator. JYP and HJK also participated in the design of the study and performed the analyses. JYK and OYK contributed to the writing of the manuscript. All authors have read and approved the final manuscript.
